# Genome-wide association study of *Mycobacterium avium* subspecies *Paratuberculosis* infection in Chinese Holstein

**DOI:** 10.1186/s12864-018-5385-3

**Published:** 2018-12-27

**Authors:** Yahui Gao, Jianping Jiang, Shaohua Yang, Jie Cao, Bo Han, Yachun Wang, Yi Zhang, Ying Yu, Shengli Zhang, Qin Zhang, Lingzhao Fang, Bonnie Cantrell, Dongxiao Sun

**Affiliations:** 1 Key Laboratory of Animal Genetics and Breeding of Ministry of Agriculture, National Engineering Laboratory of Animal Breeding, College of Animal Science and Technology, China Agricultural University, Beijing, 100193 China; 2 College of Veterinary Medicine, China Agricultural University, Beijing, 100193 China; 3 Department of Animal and Avian Sciences, University of Maryland, College Park, MD 20742 USA; 4 Department of Animal and Veterinary Sciences, University of Vermont, Burlington, VT 05405 USA

**Keywords:** GWAS, Paratuberculosis, SNP, Chinese Holstein

## Abstract

**Background:**

Paratuberculosis is a contagious, chronic and enteric disease in ruminants, which is caused by *Mycobacterium avium* subspecies *paratuberculosis* (*MAP*) infection, resulting in enormous economic losses worldwide. There is currently no effective cure for *MAP* infection or a vaccine, it is thus important to explore the genetic variants that contribute to host susceptibility to infection by *MAP*, which may provide a better understanding of the mechanisms of paratuberculosis and benefit animal genetic improvement. Herein we performed a genome-wide association study (GWAS) to identify genomic regions and candidate genes associated with susceptibility to *MAP* infection in dairy cattle.

**Results:**

Using Illumina Bovine 50 K (54,609 SNPs) and GeneSeek HD (138,893 SNPs) chips, two analytical approaches were performed, GRAMMAR-GC and ROADTRIPS in 937 Chinese Holstein cows, among which individuals genotyped by the 50 K chip were imputed to HD SNPs with Beagle software. Consequently, 15 and 11 significant SNPs (*P* < 5 × 10^− 5^) were identified with GRAMMAR-GC and ROADTDRIPS, respectively. A total of 10 functional genes were in proximity to (i.e., within 1 Mb) these SNPs, including *IL4, IL5, IL13, IRF1, MyD88, PACSIN1, DEF6, TDP2, ZAP70* and *CSF2*. Functional enrichment analysis showed that these genes were involved in immune related pathways, such as interleukin, T cell receptor signaling pathways and inflammatory bowel disease (IBD), implying their potential associations with susceptibility to *MAP* infection. In addition, by examining the publicly available cattle QTLdb, a previous QTL for *MAP* was found to be overlapped with one of regions detected currently at 32.5 Mb on BTA23, where the *TDP2* gene was anchored.

**Conclusions:**

In conclusion, we identified 26 SNPs located on 15 chromosomes in the Chinese Holstein population using two GWAS strategies with high density SNPs. Integrated analysis of GWAS, biological functions and the reported QTL information helps to detect positional candidate genes and the identification of regions associated with susceptibility to *MAP* traits in dairy cattle.

**Electronic supplementary material:**

The online version of this article (10.1186/s12864-018-5385-3) contains supplementary material, which is available to authorized users.

## Background

Paratuberculosis, also known as Johne’s disease (JD), is a contagious, chronic and enteric disease in ruminants caused by *Mycobacterium avium* subspecies *paratuberculosis* (*MAP*) [1]. Symptoms of the disease include diarrhea and weight loss that eventually leads to death. This disease has a long period of incubation [2]. In cattle, JD cannot be diagnosed until symptoms are observed because animals can have *MAP* in their systems, but not have JD. It is always difficult to determine if an animal is at risk of contracting JD by screening for *MAP*. Once a cow shows symptoms of JD, there is no treatment so the only effective means to get rid of the disease is to cull. There is also no vaccine, so farmers cannot increase resistance to JD within their herds, which causes huge economic losses. The disease can easily be spread through the farm from contact with *MAP* infected feces or milk from infected cows. Changes in herd management could help to reduce JD, but understanding resistance in cattle will allow for better management of the disease. Genomic selection for disease-resistant animals may be a promising way to increase the ability of animals to resist *MAP* infection. Exploring the genetic variants that contribute to host susceptibility to infection by *MAP* is important both for animal genetic improvement programs and for a better understanding of the underlying mechanisms of disease.

Heritability estimates in Holstein and Jersey cows for infection with *MAP* range from 0.031 to 0.283 [3–13]. By employing a case-control design, several functional genes have been reported to be associated with susceptibility to *MAP* infection in cattle. This includes *CLEC7A* [14], *IL10RA* [15], *IL12RB1*, *IL12RB2*, *IL23R*, *IFNGR2* [16], *NOD2* [17–19], *PGLYRP1* [20], *SLC11A1* [21, 22], *SP110* [23], *TLR1* [24, 25], *TLR2* [24–26] and *TLR4* [24, 25]. Multiple QTLs are located on BTA7 [27] and BTA20 [28]. These studies indicated that genetic factors contribute to the susceptibility of *MAP* infection in cattle.

Nowadays, genome-wide association study (GWAS) is a popular strategy to identify candidate genes for specified traits. Earlier GWAS studies for *MAP* infection in Holstein cattle were based on serum ELISA, milk ELISA, fecal culture test or a comprehensive test for *MAP* infection [29–37]. Various SNPs associated with susceptibly to *MAP* infection are extensively distributed across all autosomes in different Holstein cattle populations. Several candidate genes for *MAP* infection were subsequently identified, such as *EDN2*, *PRDM1*, *LAMB4*, *DLD*, *LDLRAD3*, *CACNA1B*, *TIMD4*, *ITK*, *C*, *BTN1A1* and *TDP2* [29–37]. Zare et al. reported 9 SNPs on BTA3, BTA6, BTA17 and BTA23 in the US Jersey cattle population and suggested *SLC17A1*, *UBD*, *HIVEP1*, *CCDC17*, *ZNF684*, *UBE2L3*, *UBE2K*, *FAM109A* and *FAM5C* genes as candidates for susceptibly to *MAP* infection [35]. In the present study, the objectives were to identify genetic markers and genomic regions that are associated with susceptibility to *MAP* infection by performing a GWAS in Chinese Holsteins, and to provide further molecular information for the *MAP* resistance breeding program.

## Methods

### Data description

A total of 8214 Chinese Holstein cows from 7 dairy farms belonging to the Beijing Sanyuan Dairy Farm Center were fed under the same management throughout this study. We collected a 500 μL blood sample from the caudal vein of each cow and performed a regular quarantine inspection of the farms during September 2014. Serum extracted from blood samples were stored at 4 °C until testing; within 5 days after collection. The commercially available, ELISA kit (IDEXX Laboratories, Inc., Westbrook, ME, USA) was used according to the manufacturer instructions to measure the antibody levels of each serum sample. The *MAP* status of an animal was expressed as a percentage of the sample to positive ratio (S/P) with the formula: S/P ratio = [(optical density (OD) of the sample – OD of the negative control) / (OD of positive sample – OD of the negative control)], where ≤0.45 is negative; 0.45 < S/*P* < 0.55 is suspect; S/*P* ≥ 0.55 is positive. ELISA suspect results were excluded because of their uncertainty. Out of the 8214 detected cows, 185 positive individuals (case) and 760 negative individuals (control) from 6 herds were used for GWAS. ELISA results were employed as a binary trait (0 = negative, 1 = positive).

### Genotyping

The individuals in this study were divided into 2 sub-groups for genotyping. Five hundred and thirty three cows belonging to the first sub-group were genotyped with the Illumina Bovine SNP50 BeadChip (54,609 SNPs, Illumina, San Diego, CA, USA) after extracting DNA from whole blood using routine procedures. DNA was isolated from whole blood with a commercially available kit, the DP318 Blood DNA Kit (Tiangen Biotech Co., China). The DNA of the remaining 412 cows in the second sub-group were extracted from hair by GeneSeek with QIAamp® DNA Mini Kit (QIAGEN Inc., Valencia, CA, USA) and then genotyped with the GeneSeek Genomic Profiler HD v2 (138,893 SNPs, GeneSeek, Lincoln, NE, USA). The genotype data were deposited in the Additional files (Additional files 1 and 2).

### Imputation and quality control

To make full use of SNPs originating from the GeneSeek Genomic Profiler HD v2 (GeneSeek), individuals genotyped by the Illumina Bovine SNP50 BeadChip were imputed to GeneSeek Genomic Profiler HD v2. Imputation was performed using BEAGLE 3.3.2 [38] default options. Allelic R^2^ was estimated as an indicator of imputation accuracy based on the genotype probabilities.

To further evaluate imputation accuracy, we randomly selected 100 cows genotyped by the high-density chip, obtained the common SNPs between the two panels, and masked genotypes of SNPs left. We classified this small subset of cows as the study population, while the remaining cows genotyped by the high-density panel were classified as the reference population. After imputation, we compared the imputed and masked actual genotypes of the selected 100 cows to calculate the percentage of genotypes that are consistent between them.

Then we implemented PLINK [39] and removed SNPs with call rates < 95%, minor allele frequencies < 0.01, a deviation from Hardy-Weinburg equilibrium (HWE) *P* values < 10^− 6^ and > 5% missing genotypes. A dataset containing 109,607 SNPs and 937 animals (182 cases and 755 controls) was used for further analysis. All SNP positions were determined according to the *Bos taurus* UMD 3.1 assembly [40].

### Population stratification

Differences in allele frequencies between subpopulations of admixed populations can lead to false association in a GWAS [41]. In order to determine whether stratification exists in our study population, a principle component analysis (PCA) was performed by GCTA 1.24 [42] and results were visualized by R 3.3.1 [43].

### GWAS

#### GRAMMAR-GC

We performed a GWAS using the Genome-wide Rapid Association using Mixed Model and Regression-Genomic Control (GRAMMAR-GC) approach [44, 45], a single-marker method implemented within the GenABEL package [46] for R [43]. This approach can account for the potential population structure and infer relationships using SNP data without pedigree information. There have been multiple GWAS studies in cattle that utilized this approach [31, 34–37]. GRAMMAR-GC is comprised of three steps that use the regression of phenotypes on the genotypes of individuals for one SNP at a time. First, to account for familial dependence among individuals, phenotypes were corrected by conducting a polygenic analysis using a genomic kinship matrix based on the SNP genotypes. Residuals from the polygenic analysis were then used as dependent quantitative traits for association analysis of each SNP with a linear regression model. Finally, genomic control (GC) was applied to correct the test statistic using the genomic inflation factor (λ), which is the regression coefficient of the observed statistic on the expected statistic. We performed an association test for each SNP based on the following linear mixed model:

$$ \mathrm{y}=\mathrm{W}\upalpha +\mathrm{x}\upbeta +\mathrm{u}+\upvarepsilon, $$

where **y** is the liability vector for case/control observations; **W** is a matrix of covariates (fixed effects that contain herd and parity); **α** is a vector of the corresponding coefficients including the intercept; **x** is a vector of genotypes of a marker at the locus tested; **β** is the effect size of the marker; **u** is a vector of random polygenic effects with a covariance structure as **β**~N (0, V_g_), V_g_ is the polygenic additive variance; **ε** is a vector of residual errors with **ε**~N (0, **I**V_e_), **I** is the identity matrix, and V_e_ is the residual variance component.

In general, for GRAMMAR-GC, the value ${T}^2/\widehat{\zeta}$ of each SNP with one-degree freedom is compared with ${\chi}_1^2$ to determine whether the locus is significantly associated with the trait. Here ${T}_k^2={\widehat{\beta}}_k^2/\operatorname{var}\left(\widehat{\beta_k}\right)$, where $\widehat{\beta_k}$ is the effect of the *k*^*th*^ SNP. The deflation factor ζ is estimated as ζ *= median* (${T}_1^2,{T}_2^2,\dots, {T}_k^2$)/0.456.

#### ROADTRIPS

A second GWAS approach was implemented with ROADTRIPS 2.0 [47]. An important advantage of ROADTRIPS 2.0 is that it can analyze data with pedigree information and population admixture simultaneously. Based on the genome-wide SNP data, an empirical covariance matrix was constructed to adjust for potential population admixture and relatedness among individuals and maintain the advantage of utilizing known pedigree information when available. The ROADTRIPS 2.0 test statistic based on ${\chi}_1^2$ distribution for each SNP takes the form:

$$ \frac{{\left({\mathbf{V}}^T\mathbf{Y}\right)}^2}{{\widehat{\sigma}}^2{\mathbf{V}}^T\widehat{\boldsymbol{\Psi}}\mathbf{V}}\sim {\chi}_1^2 $$

Here **Y** = (*Y*_1_, *Y*_2_, …, *Y*_n_)^*T*^, is genotype vector at a test SNP for n individuals (coded using an allelic coding). **V** is a vector of length n coding for phenotype information (disease status) and known relationships.${\widehat{\sigma}}^2\widehat{\boldsymbol{\Psi}}$ is an estimate of the null variance/covariance matrix of **Y**.${\widehat{\sigma}}^2$ is an estimate of Var(Y) in an outbred population and$\widehat{\boldsymbol{\Psi}}$ is an estimated matrix used to simultaneously adjust for unknown relatedness/pedigree relationship errors and population stratification.

ROADTRIPS 2.0 provides three association tests named RM test, Rχ test and RW test. According to the authors’ recommendation, the RM test is the most powerful among the three tests when pedigree information is available. Compared with the Rχ test and the RW test, the RM test can use the phenotypic information of individuals with missing genotypes provided that they have a genotyped relative at the tested marker. Considering the features of the RM test and the data structure of this study being based on a corrected pedigree, we adopted the RM test for association analysis. *P* values of SNPs were derived from an asymptotic chi-square distribution with 1 degree of freedom. In addition, the fixed effects used here was the same as above.

Following the suggestion of the Welcome Trust Case Control Consortium [48], two *P* value thresholds of 5 × 10^− 7^ and 5 × 10^− 5^ were considered as genome-wide “strong” and “moderate” association respectively.

### Gene contents and functional annotation

Using BioMart in the Ensembl database (Ensembl Genes 92), genes within 1 Mb of the significant SNPs were retrieved based on the UMD 3.1 assembly. To provide insight into the functional enrichment of genes identified, we carried out GO (Gene Ontology) and Pathway analysis using KOBAS 3.0 [49]. KOBAS annotates a set of genes with putative pathways and disease relationships by mapping to genes with a known annotation. In addition, we compared the regions within 1 Mb of the significant SNPs with the reported cattle to QTLs for JD tolerance and *MAP* susceptibility in the Animal QTL database (http://www.animalgenome.org/cgi-bin/QTLdb/index) [50].

## Results

### Imputation accuracy

After imputation, we discarded SNPs with allelic R^2^ < 0.85 and found an average allelic R^2^ of 96.7% for imputed genotypes. Then we took a small subset including 100 cows genotyped by the high-density chip for calculating the imputation accuracy. Finally, the percentage of consistent genotypes was 97.03%, which suggested a high accuracy of imputation.

### GWAS based on GRAMMAR-GC

**Fig. 1:**
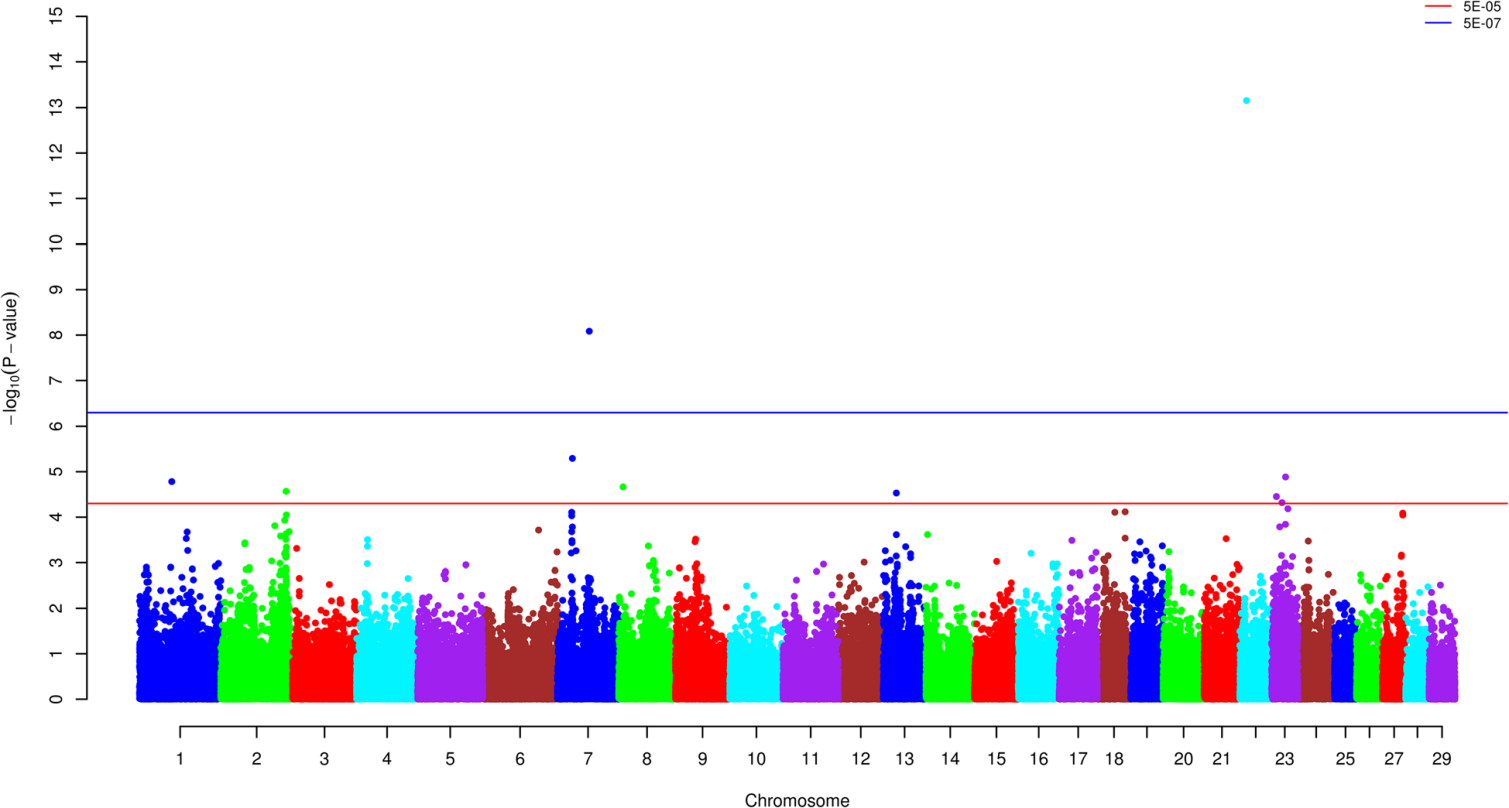
Manhattan plot of genome-wide SNPs obtained using GRAMMAR-GC. Each dot represents the result from the test of association for a single SNP. Minus log10 of the *P* value is indicated on the y-axis and map location of the SNP is indicated on the x-axis. Thresholds represent *P* values of 5E-05 and 5E-07 for moderate and strong associations, respectively

With GCTA 1.24, a slight population substructure was revealed (Additional file 3: Figure S1). The inflation factor (λ), estimated to be 0.9399 (SE = 0.0002), indicates population substructure was a minor issue and that our results can be accepted for further analysis. The GC-corrected *P* values for the majority of SNPs corresponded well to the expected *P* values under the null hypothesis of no association. However, a few departures which mean the *P* values of these SNPs were higher than the expected *P* values under the null hypothesis indicated associations with the trait being studied (Additional file 4: Figure S2). As shown in Tables 1, 2 SNPs passed the strong association threshold and 13 SNPs passed the moderate threshold (Fig. 1).

**Table 1:** Results of GRAMMAR-GC genome-wide association analysis for susceptibility to *MAP* infection (df = 1)

SNP	BTA	Position	*P* value
BovineHD2200003382	22	11,359,993	1.13E-14
BovineHD0700017491	7	60,947,866	2.76E-09
BovineHD0700007000	7	25,403,106	2.54E-06
BovineHD2300007824	23	28,173,531	6.90E-06
BTB-00030699	1	61,806,466	8.85E-06
BovineHD0800002130	8	6,659,432	1.18E-05
BovineHD0200036516	2	125,910,848	1.49E-05
Hapmap44402-BTA-73818	13	27,398,154	1.64E-05
BovineHD4100015938	23	8,975,441	1.98E-05
ARS-BFGL-BAC-29490	23	20,166,517	2.75E-05
BovineHD2300009447	23	32,516,000	3.82E-05
ARS-BFGL-NGS-36626	18	54,052,117	4.50E-05
BovineHD0700006647	7	24,259,310	4.62E-05
BovineHD1800010086	18	33,293,455	4.62E-05
BovineHD2700011748	27	40,498,309	4.83E-05

**Table 2:** Results of ROADTRIPS genome-wide association analysis for susceptibility to *MAP* infection (df = 1)

SNP	BTA	Position	*P* value
BTB-01281916	3	71,760,025	1.21E-08
Hapmap49590-BTA-38619	16	35,765,411	4.37E-08
ARS-BFGL-NGS-25380	11	2,260,123	1.98E-07
BTB-00452217	11	2,983,521	2.81E-07
BTA-79476-no-rs	7	60,933,059	9.71E-07
BovineHD0700006447	7	23,495,415	2.10E-06
BovineHD0200035709	2	123,174,023	7.90E-06
ARS-USDA-AGIL-chr6–117,920,790-000733	6	117,920,790	1.87E-05
BovineHD2400014963	24	52,857,741	3.33E-05
Hapmap39753-BTA-50189	20	28,186,842	4.80E-05
BovineHD1300015832	13	55,734,067	4.92E-05

### GWAS based on ROADTRIPS

**Fig. 2:**
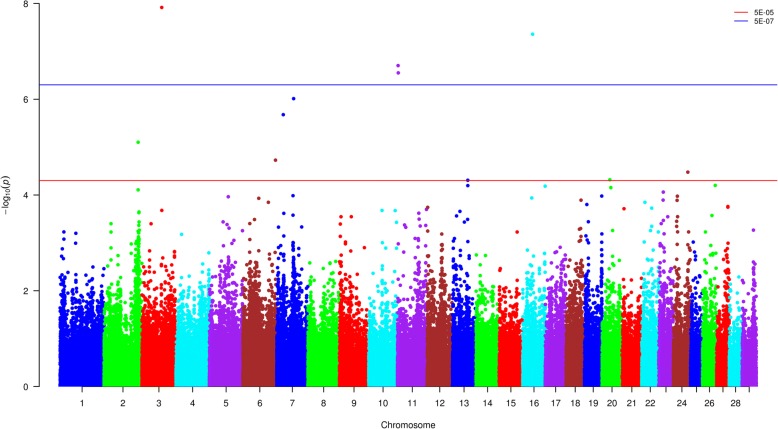
Manhattan plot of genome-wide SNPs obtained using ROADTRIPS. Each dot represents the result from the test of association for a single SNP. Minus log10 of the *P* value is indicated on the y-axis and map location of the SNP is indicated on the x-axis. Thresholds represent *P* values of 5E-05 and 5E-07 for moderate and strong associations, respectively

RM test was implemented for the association analysis. As shown in Additional file 5: Figure S3, the *P* values for the majority of SNPs exhibited a good correspondence to the expected values with a limited number of SNPs indicating their associations with the studied trait. In total, 4 and 7 SNPs passed the threshold of strong and moderate association, respectively (Fig. 2, Table 2).

### Gene contents and functional annotation

There were 15 significant SNPs detected by GRAMMAR-GC in total. Utilizing BioMart in the Ensembl database (Ensembl Genes 92), we obtained the 232 IDs for genes located within or overlapped with the regions nearby these SNPs (< 1 Mb) (Additional file 6: Table S1). Based on the 11 significant SNPs detected by ROADTRIPS, 123 functional gene IDs were identified (Additional file 7: Table S2). After the combination of results, a total of 343 genes were obtained, including 283 protein-coding genes, 21 miRNA genes, 6 pseudogenes, 12 snRNA, 15 snoRNA, 4 rRNA and 2 miscRNA (Additional file 8: Table S3).

GO and Pathway analysis were performed by KOBAS 3.0 to determine the biological functions of the 343 genes. Finally, 348 significant GO terms were detected, including those related to immune response (*P* < 0.05), such as immune response-regulating signaling pathway, regulation of leukocyte proliferation and immune response-activating signal transduction. Fifteen significant pathways were found, including those related to immune responses (*P* < 0.05) such as autoimmune thyroid disease and enrichment of the interleukin signaling pathway (Additional file 9: Table S4). In addition, T cell receptor signaling pathway [51] and inflammatory bowel disease (IBD) were detected but not significant.

### Quantitative traits locus overlapped with SNPs

Until now, 6 JD tolerance and 161 *MAP* susceptibility QTLs have been reported in the cattle QTL database (http://www.animalgenome.org/cgi-bin/QTLdb/BT/index). After comparing these QTLs with the regions within 1 Mb of the 26 significant SNPs, 2 QTLs identified before [30, 37] located in BTA23 (~ 32.5 Mb) for *MAP* susceptibility were found. This implies the functional genes, such as *TDP2* (tyrosyl-DNA phosphodiesterase 2) around these SNPs are likely candidates for *MAP* susceptibility traits.

## Discussion

There have been multiple GWASs conducted in different cattle population [29–37], and some candidate loci and genes have been identified. Although these studies found evidence of genomic regions associated with *MAP* infection, the consistency was not high. The genomic regions and genes regarded as candidates for the target traits were variable among previous studies. The difference between genomic regions identified by different studies is because of different trait definitions except for statistical methodologies [52]. There were four main definitions for infection cases in previous studies: ELISA positive [27, 31, 33], fecal culture positive [29], tissue culture positive [29] and comprehensive testing. Comprehensive testing includes tissue culture positive and fecal culture positive [29], ELISA positive or fecal positive [30], and ELISA positive or tissue culture positive [34].

We found 15 SNPs passing the threshold (5 × 10^− 5^) using GRAMMAR-GC with BTA23 owning the most SNPs. The most significant SNP, BovineHD2200003382 (*P* = 1.13E-14) was identified on BTA22 (Table 1). Similarly, 11 SNPs passed the threshold (5 × 10^− 5^) using ROADTRIPS with the most significant SNP, BTB-01281916 (*P* = 1.21E-08) identified on BTA3 (Table 2). There was no common SNP sharing between these two methods because of some possible reasons. Firstly, different computational principles can cause different significant SNPs. It is common that the discrepancy caused by this reason, such as the report of Alpay et al. [36] and Sallam et al. [37]. Both ROADTRIPS and GRAMMAR-GC can correct sample structure. The ROADTRIPS program uses the quasi-likelihood methods (implemented in the MQLS and similar statistics), to obtain known kinship coefficients, which then together with the empirical covariance matrix estimated from genomic data to correct for known and unknown relatedness and population structure [47]. Instead of pedigree, GRAMMAR-GC program uses genomic kinship matrix estimated through genomic marker data to adjust for average allele sharing or relatedness among sample individuals and thus remove genetic stratification [44, 45].

Secondly, JD is affected by multiple genetic loci and SNPs identified using different methods were polygenic in present study. Those SNPs with large effect may be captured more easily by multiple GWAS methods. In addition, the size of study population may cause discrepancy. The more individuals, the higher the accuracy of GWAS result. So it seems normal that different methods identifying different SNPs in present study. Thus, it seems normal that different methods identify different SNPs.

While no SNPs were identified by two methods, but the SNPs between the two methods were located close to each other on the same chromosome. For example on BTA2 ~ 2.74 Mb was found between BovineHD0200036516 (GRAMMAR-GC) and BovineHD0200035709 (ROADTRIPS), on BTA7 ~ 14.81 Kb was found between BovineHD0700017491 (GRAMMAR-GC) and BTA-79476-no-rs (ROADTRIPS), on BTA7 ~ 1.91 Mb was found between BovineHD0700007000 (GRAMMAR-GC) and BovineHD0700006447 (ROADTRIPS) and ~ 0.76 Mb was found between BovineHD0700006647 (GRAMMAR-GC) and BovineHD0700006447 (ROADTRIPS). Combining the results of these two methods, 26 significant SNPs were obtained. The most SNPs were found on BTA7 followed by BTA23.

Among the 26 significant SNPs detected by two methods, BovineHD0700006447 (23.5 Mb) located on BTA7 in this study was close to SNPs detected by Pant et al. (20.6 Mb ~ 22.3 Mb) [27]. Genes nearby this SNP within less than 1 Mb were *IL4*, *IL5*, *IL13* and *IRF1*. The genes, *IL4* (interleukin 4), *IL5* and *IL13* are type 2 cytokines, that may be the predominant cytokines produced by CD4+ and other T cells in lymph nodes during the subclinical infection of *MAP* [53]. As previously reported [54–56], in the clinical infection, the bovine *MAP* infection disease was characterized by a gradual shift in the immune responses from cell-mediated immune response to antibody mediated immune response while *IL4*, *IL5* and *IL13* can promote the T_h_2 antibody-mediated immune response. Therefore, these three genes might play important roles in the pathogenesis of the disease [27]. *IRF1*, interferon regulatory factor 1, plays an important role in many immune responses including the Type 1 (T_h_1) cell-mediated immune response. Cell mediated immunity is an important host defense mechanism against intracellular pathogens including *MAP* [57]. In addition, it can regulate the expression of many immune genes such as *IL6*, *IL12B*, and inducible nitric oxide synthase (NOS2) that function in the pathogenesis of human IBD [58–60].

The most significant SNP, BovineHD2200003382 detected by GRAMMAR-GC was located at 11.3 Mb on BTA22. Genes within 1 Mb of this location includes *MyD88* (myeloid differentiation primary response gene 88) which encodes a cytosolic adapter protein that plays a central role in the innate and adaptive immune response. MyD88 functions as an essential signal transducer in the interleukin-1 and Toll-like receptor signaling pathways [61].

SNP BovineHD4100015938 on BTA23 (8.98 Mb) was close to ARS-BFGL-NGS-109956 (7.84 Mb) and ARS-BFGL-NGS-115177 (7.87 Mb) reported by Zare et al. [35]. The genes near to these two SNPs included *PACSIN1* and *DEF6* that are related to immune response. *PACSIN1* (protein kinase C and casein kinase substrate in neurons 1), belonging to a family of cytoplasmic phosphoproteins, participates in the regulation of endocytosis [62] and regulates the TLR7/9-mediated type I interferon response in plasmacytoid dendritic cells [63]. *DEF6* (DEF6, guanine nucleotide exchange factor) is a guanine nucleotide exchange factor (GEF) for RAC (MIM 602048) and CDC42 (MIM 116952) that are highly expressed in B and T cells [64]. SNP BovineHD2300009447 (32.5 Mb) located on BTA23 was very close to ARS-BFGL-NGS-1938 (32.6 Mb) reported by Zare et al. in Jersey cattle [35], and close to ss105264543 (33.6 Mb) reported by Minozzi et al. in Holstein cattle [34]. Gene within 1 Mb of this region was *TDP2* (tyrosyl-DNA phosphodiesterase 2). This gene encodes a member of a superfamily of divalent cation-dependent phosphodiesterases. The encoded protein associates with CD40, tumor necrosis factor (TNF) receptor-75 and TNF receptor associated factors (TRAFs) that inhibits nuclear factor-kappa-B activation. In addition, TDP2 has sequence and structural similarities with APE1 endonuclease, which is involved in both DNA repair and the activation of transcription factors [65].

Interleukin, T cell receptor signaling pathway and inflammatory bowel disease (IBD) pathways are related to immune or inflammatory response. Two genes including *ZAP70* and *SRF*, except for *IL4*, *IL5*, *IL13* and *MyD88* stated above, were involved in the immune biological processes. *ZAP70* (zeta chain of T-cell receptor associated protein kinase 70) encodes an enzyme belonging to the protein tyrosine kinase family that plays a role in T-cell development and lymphocyte activation. This enzyme, phosphorylated on tyrosine residues upon T-cell antigen receptor (TCR) stimulation, functions in the initial step of TCR-mediated signal transduction in combination with the Src family kinases, Lck and Fyn and plays an essential role in the process of thymocyte development [66]. In addition, mutations in this gene cause selective T-cell defect, a severe combined immunodeficiency disease characterized by a selective absence of CD8-positive T-cells [67]. Leite et al. investigated the expression of *ZAP70* in cows naturally infected with *MAP* and revealed that the surface expression of *ZAP70* was decreased in CD4+ T cells of both subclinical and clinical animals indicating a change in T cell phenotype with disease state [68]. *CSF2* (colony stimulating factor 2), also known as *CSF* and *GMCSF*, encodes a cytokine that controls the production, differentiation, and function of granulocytes and macrophages. This gene has been localized to a cluster of related genes at chromosome region 5q31 that are known to be associated with interstitial deletions in the 5q- syndrome and acute myelogenous leukemia. Other genes in the cluster include those encoding interleukins 4, 5, and 13 [69].

Furthermore, we found a region nearby the BovineHD2300009447 (BTA23, 32.5 Mb) overlapped with one QTL associated with *MAP* susceptibility. Combing this information with related genes found above, the 32 ~ 33 Mb region of BTA23 may be a case of a genomic region associated with *MAP* infection, which corresponds to the report of Zare et al. [35].

The present study focused on the potential function of 10 candidate genes. Future analysis is necessary to investigate the biological processes and molecular mechanism of these genes to anchor immune alterations and possible triggers that result in clinical paratuberculosis.

## Conclusions

We performed a case-control GWAS for *MAP* infection in Chinese Holstein cattle using two statistical approaches, GRAMMAR-GC and ROADTRIPS. Twenty-six significant SNPs located on 15 chromosomes were detected based on data after imputation. Ten genes within less than 1 Mb of these SNPs were involved in immune response pathways, implying their potential associations with susceptibility to *MAP*. These genes included *IL4, IL5, IL13, IRF1, MyD88, PACSIN1, DEF6, TDP2, ZAP70* and *CSF2*. By examining the QTLdb, the 32 ~ 33 Mb region of BTA23 may be a genomic region associated with *MAP* infection.

## Additional files

Additional file 1: MAP file of SNP data. (MAP 3577 kb)

Additional file 2: PED file of SNP data. (PARTIAL 776 kb)

Additional file 3: **Figure S1.** PCA plot based on SNP data. (TIFF 175 kb)

Additional file 4: **Figure S2.** Q-Q plot based on SNP data using GRAMMAR-GC. (TIFF 175 kb)

Additional file 5: **Figure S3.** Q-Q plot based on SNP data using ROADTRIPS. (TIFF 179 kb)

Additional file 6: **Table S1.** The features of genes based on GRAMMAR-GC. (XLSX 20 kb)

Additional file 7: **Table S2.** The features of genes based on RODATRIPS. (XLSX 15 kb)

Additional file 8: **Table S3.** The features of genes based on GRAMMAR-GC and RODATRIPS. (XLSX 26 kb)

Additional file 9: **Table S4.** Functional enrichment of GO and Pathway analysis of 343 genes. (XLSX 32 kb)

## Abbreviations

GC: Genomic control
GRAMMAR-GC: Genome-wide Rapid Association using Mixed Model and Regression-Genomic Control
GWAS: Genome-wide association study
JD: Johne’s disease
MAP: Mycobacterium avium subspecies Paratuberculosis
PCA: Principle component analysis
QQ: Quantile-quantile
QTL: Quantitative trait locus
SNP: Single nucleotide polymorphisms
