# Supplementary figures and images for: Genome-wide association study of Mycobacterium avium subspecies Paratuberculosis infection in Chinese Holstein
Source: BMC Genomics. 2018 Dec 27;19:972. doi: 10.1186/s12864-018-5385-3 (PMC6307165; doi:10.1186/s12864-018-5385-3)

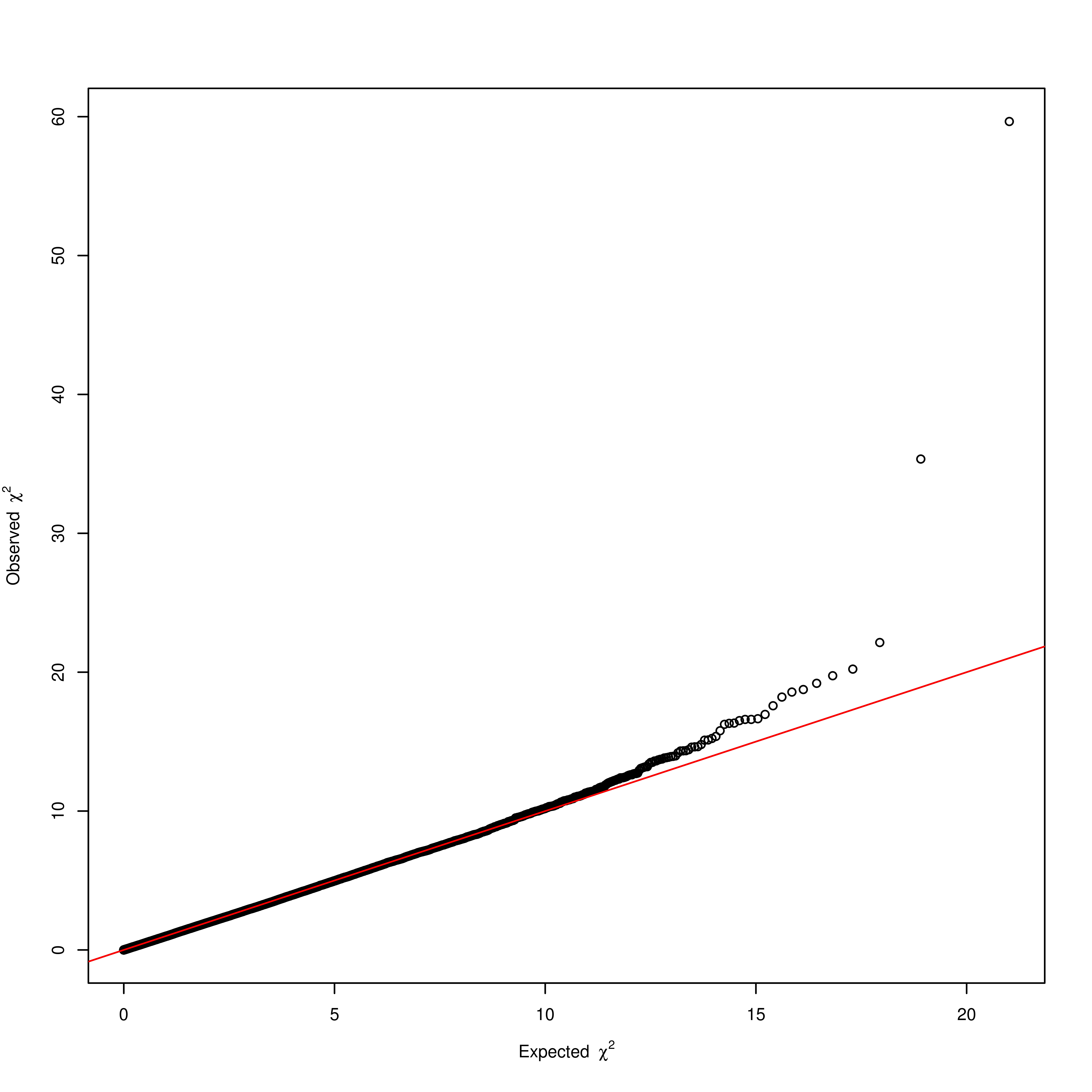

Supplement: Supplementary file 3 — Figure S1. PCA plot based on SNP data. (TIFF 175 kb) [file 12864_2018_5385_MOESM3_ESM.tiff]

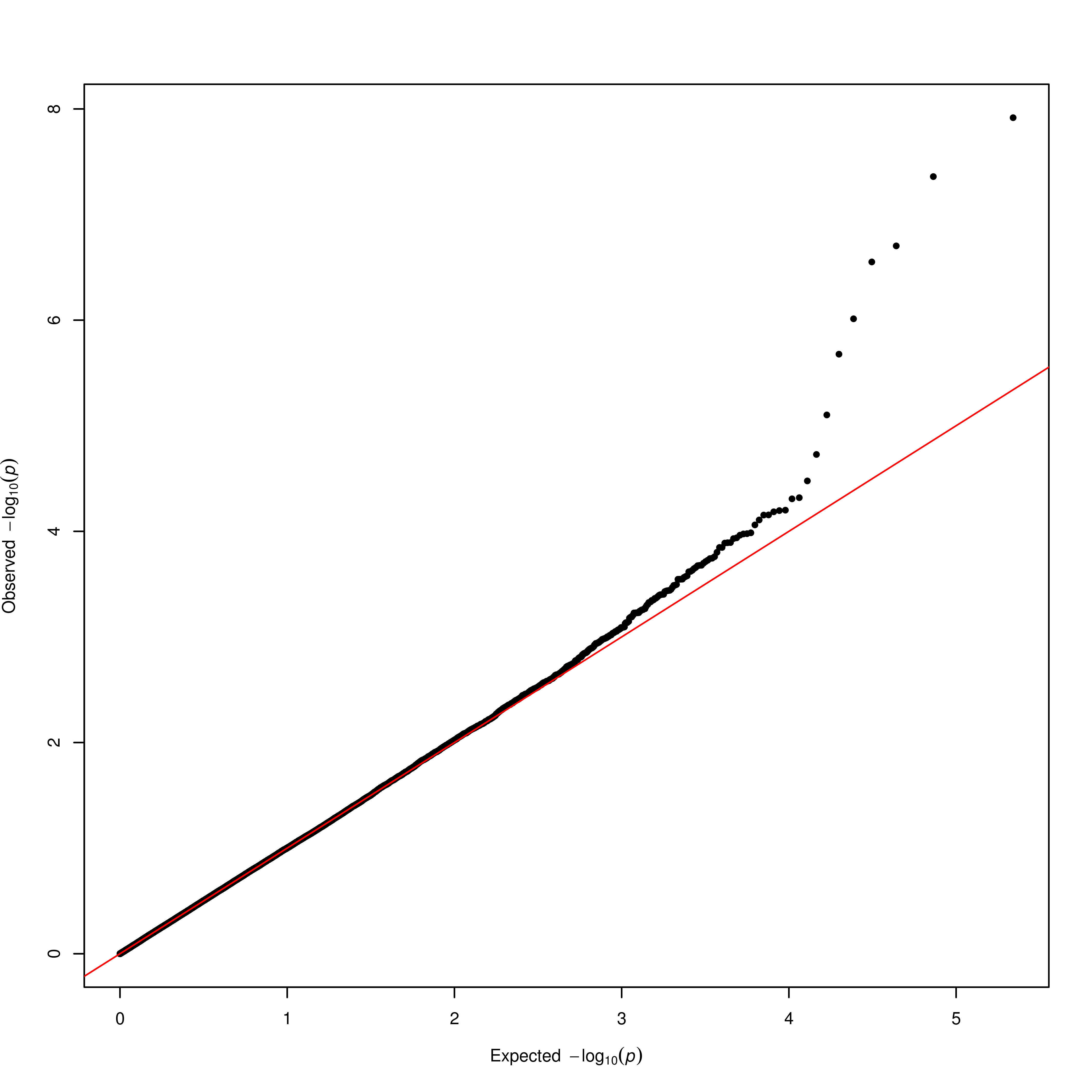

Supplement: Supplementary file 5 — Figure S3. Q-Q plot based on SNP data using ROADTRIPS. (TIFF 179 kb) [file 12864_2018_5385_MOESM5_ESM.tiff]
